# *Clostridium thermocellum* DSM 1313 transcriptional responses to redox perturbation

**DOI:** 10.1186/s13068-015-0394-9

**Published:** 2015-12-12

**Authors:** Kyle Sander, Charlotte M. Wilson, Miguel Rodriguez, Dawn M. Klingeman, Thomas Rydzak, Brian H. Davison, Steven D. Brown

**Affiliations:** Bredesen Center for Interdisciplinary Research and Graduate Education, University of Tennessee, Knoxville, TN 37996 USA; BioEnergy Science Center, Oak Ridge National Laboratory, Oak Ridge, TN 37831 USA; Department of Chemical and Biomolecular Engineering, University of Tennessee, Knoxville, TN 37996 USA; Biosciences Division, Oak Ridge National Laboratory, Oak Ridge, TN 37831 USA

**Keywords:** *Clostridium thermocellum* DSM 1313, Microarray, Transcriptomics, Methyl viologen, Chemostat, Redox, Sulfate, GS-GOGAT, Hydrogenase

## Abstract

**Background:**

*Clostridium thermocellum* is a promising consolidated bioprocessing candidate organism capable of directly converting lignocellulosic biomass to ethanol. Current ethanol yields, productivities, and growth inhibitions are industrial deployment impediments for commodity fuel production by this bacterium. Redox imbalance under certain conditions and in engineered strains may contribute to incomplete substrate utilization and may direct fermentation products to undesirable overflow metabolites. Towards a better understanding of redox metabolism in *C. thermocellum*, we established continuous growth conditions and analyzed global gene expression during addition of two stress chemicals (methyl viologen and hydrogen peroxide) which changed the fermentation redox potential.

**Results:**

The addition of methyl viologen to *C. thermocellum* DSM 1313 chemostat cultures caused an increase in ethanol and lactate yields. A lower fermenter redox potential was observed in response to methyl viologen exposure, which correlated with a decrease in cell yield and significant differential expression of 123 genes (log_2_ > 1.5 or log_2_ < −1.5, with a 5 % false discovery rate). Expression levels decreased in four main redox-active systems during methyl viologen exposure; the [NiFe] hydrogenase, sulfate transport and metabolism, ammonia assimilation (GS-GOGAT), and porphyrin/siroheme biosynthesis. Genes encoding sulfate transport and reduction and porphyrin/siroheme biosynthesis are co-located immediately downstream of a putative *iscR* regulatory gene, which may be a cis-regulatory element controlling expression of these genes. Other genes showing differential expression during methyl viologen exposure included transporters and transposases.

**Conclusions:**

The differential expression results from this study support a role for *C.* *thermocellum* genes for sulfate transport/reduction, glutamate synthase-glutamine synthetase (the GS-GOGAT system), and porphyrin biosynthesis being involved in redox metabolism and homeostasis. This global profiling study provides gene targets for future studies to elucidate the relative contributions of prospective pathways for co-factor pool re-oxidation and *C.* *thermocellum* redox homeostasis.

**Electronic supplementary material:**

The online version of this article (doi:10.1186/s13068-015-0394-9) contains supplementary material, which is available to authorized users.

## Background

*Clostridium thermocellum* natively expresses enzymes to both deconstruct lignocellulosic biomass and ferment cellulose into ethanol, making it a candidate biocatalyst for consolidated bioprocessing (CBP). *C.* *thermocellum* hydrolyzes lignocellulosic biomass rapidly and efficiently using an elaborate enzyme system in the form of free and cell bound multi-enzyme cellulolytic complexes called cellulosomes [1, 2]. Although its hydrolysis machinery is among the fastest and most effective known [3], its fermentative metabolism results in yields and productivities too low for cost-effective industrial lignocellulosic ethanol production [2, 4].

In addition to ethanol, *C. thermocellum* natively produces acetate, lactate, formate, and hydrogen. Efforts to eliminate these undesirable products [5–7], in conjunction with metabolic engineering of lignocellulosic substrates, have been met with higher ethanol yields [8]. Another limitation to ethanol yield and productivity is a presumed ‘overflow’ metabolism by which *C. thermocellum* makes a number of other products, a phenomenon that seems to be exacerbated when fermentative metabolic pathways that reoxidize redox co-factors are eliminated or when substrate loadings are relatively high [9–11]. *C. thermocellum* metabolism is affected by the addition of exogenous fermentation products [12] and inhibitor chemicals [13] as well as other environmental perturbations. These responses were seen through altered end-product distributions, O/R balances, and inhibited substrate uptake. Further, metabolic changes that originate at the level of sensing and transcription have been observed in response to different physical and chemical perturbations [14–17]. The mechanisms by which *C.* *thermocellum* senses, regulates, and balances redox status remain poorly understood and a deeper understanding may inform future metabolic engineering efforts. The potential inability of various engineered and wild-type strains to sufficiently reoxidize redox co-factors and possible cellular redox imbalance from overly reduced co-factor pools is an area of biotechnological interest.

Redox metabolism has been studied in many organisms capable of carrying out a variety of redox reactions. One method to examine redox-related metabolism is to observe gene expression responses to an altered redox environment [18, 19]. Other studies have employed comparative genomics approaches [20], *rex* regulatory gene deletion studies [21, 22], or high-throughput genetic approaches such as rapid transposon liquid enrichment sequencing (TnLE-seq) under stress conditions [23] to investigate physiological and regulatory responses. Such studies give insights into not only transcriptional responses, but also regulons and signaling responses to such environments. After methyl viologen exposure, *Clostridium acetobutylicum* showed decreased expression of solvent-producing genes, as well genes involved in sulfate and iron transport, while butanol synthesizing genes showed increased transcription concomitant with a much higher butanol/acetone ratio [19]. Fermentation and metabolite analysis of *Clostridium cellulolyticum* implicated high NADH/NAD^+^ ratio, and low pyruvate:ferredoxin oxidoreductase activity causing limited fermentative metabolism and production of overflow products [24, 25]. Hence, studies into redox metabolism have the potential to not only provide fundamental insights but also have potential to advance applied goals.

In this study, we established *C. thermocellum* steady-state chemostat cultures and investigated redox processes after perturbing environmental conditions through separate additions of two redox-active chemicals; methyl viologen and hydrogen peroxide. Fermentative changes were observed and transcriptional responses of *C. thermocellum* DSM 1313 were studied using DNA microarray analyses.

## Results and discussion

### Preliminary batch experiments with methyl viologen

Preliminary batch growth indicated methyl viologen reduction in vivo required viable metabolically active cellular biomass. A change to blue coloration was used as an indication of methyl viologen reduction. To assess methyl viologen reduction, abiotic medium, autoclave killed cells (in medium or water), live growing cells, and cell-free spent medium, each containing 150 mg/L methyl viologen, were incubated in *C. thermocellum* growth conditions overnight. Blue coloration was only observed after incubation of cells from log-phase or stationary-phase cultures medium containing cellobiose and methyl viologen. Blue coloration was not observed after 5 days of incubation in fresh medium, spent medium, autoclaved cells (in medium or resuspended in water), cells (log phase or stationary phase) resuspended in water, after 5 days of incubation at 55 °C. Preliminary batch fermentations were conducted to estimate the appropriate methyl viologen concentration to introduce into the carbon-limited chemostats (Additional file 1). A decrease in growth rate of batch cultures was seen in cultures containing initial loadings of methyl viologen of 150 ug/mL and higher. Consistent with an earlier study [26], an increase in end-point ethanol productivity was observed in cultures containing methyl viologen (Additional file 1) and at a final concentration of 150 mg/L was used in chemostat studies. Methyl viologen has been shown to inhibit *Clostridium butyricum* hydrogen production during glycerol fermentation [27]; however, in preliminary *C. thermocellum* cellobiose batch studies specific end-point H_2_ productivity increased with methyl viologen.

### Chemostat response to methyl viologen addition

**Fig. 1:**
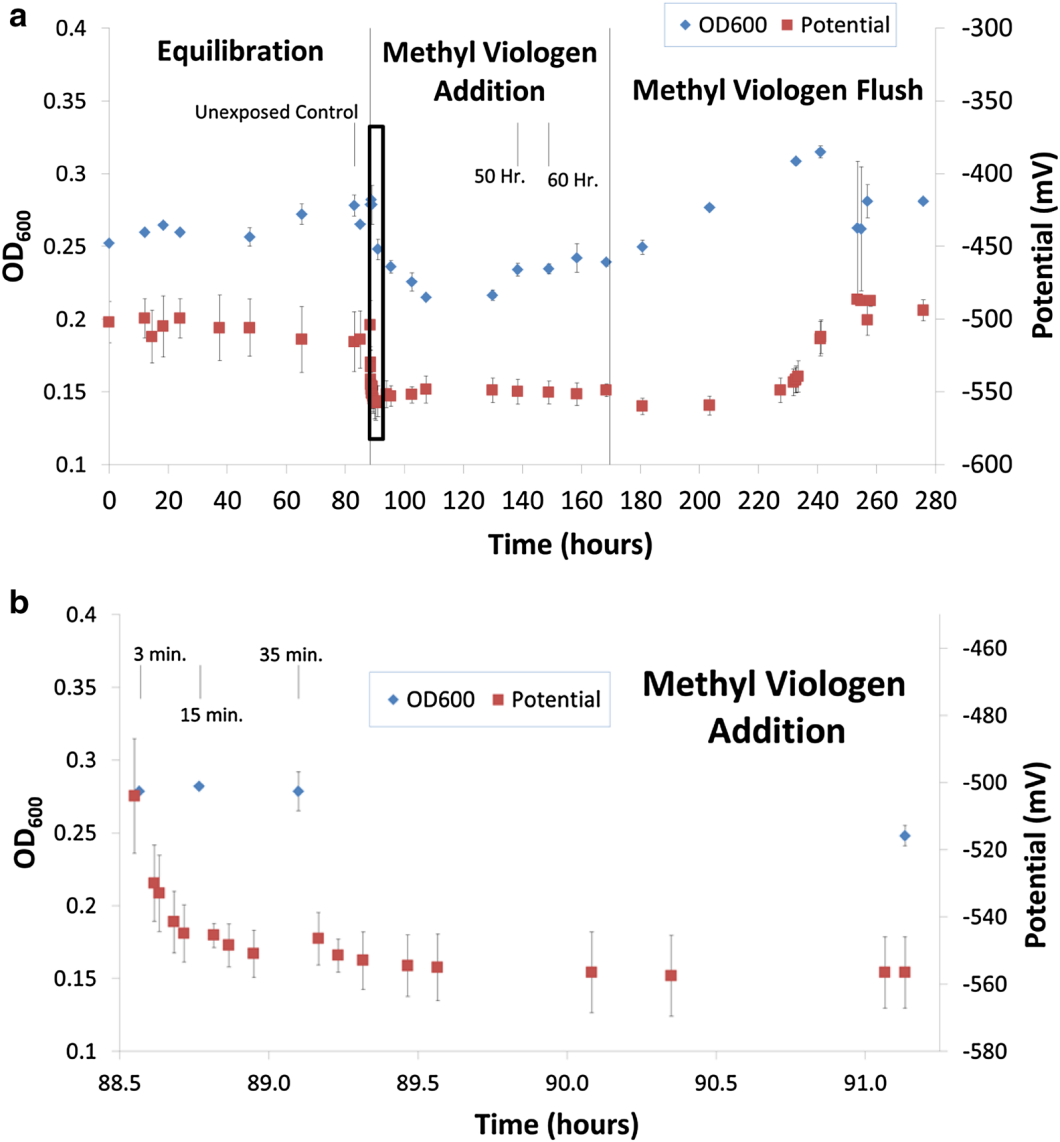
Chemostat growth and redox potential before, during, and after methyl viologen addition. **a** Over 280 h and **b** for detailed view of *boxed region* indicated in *panel* **a**

**Fig. 2:**
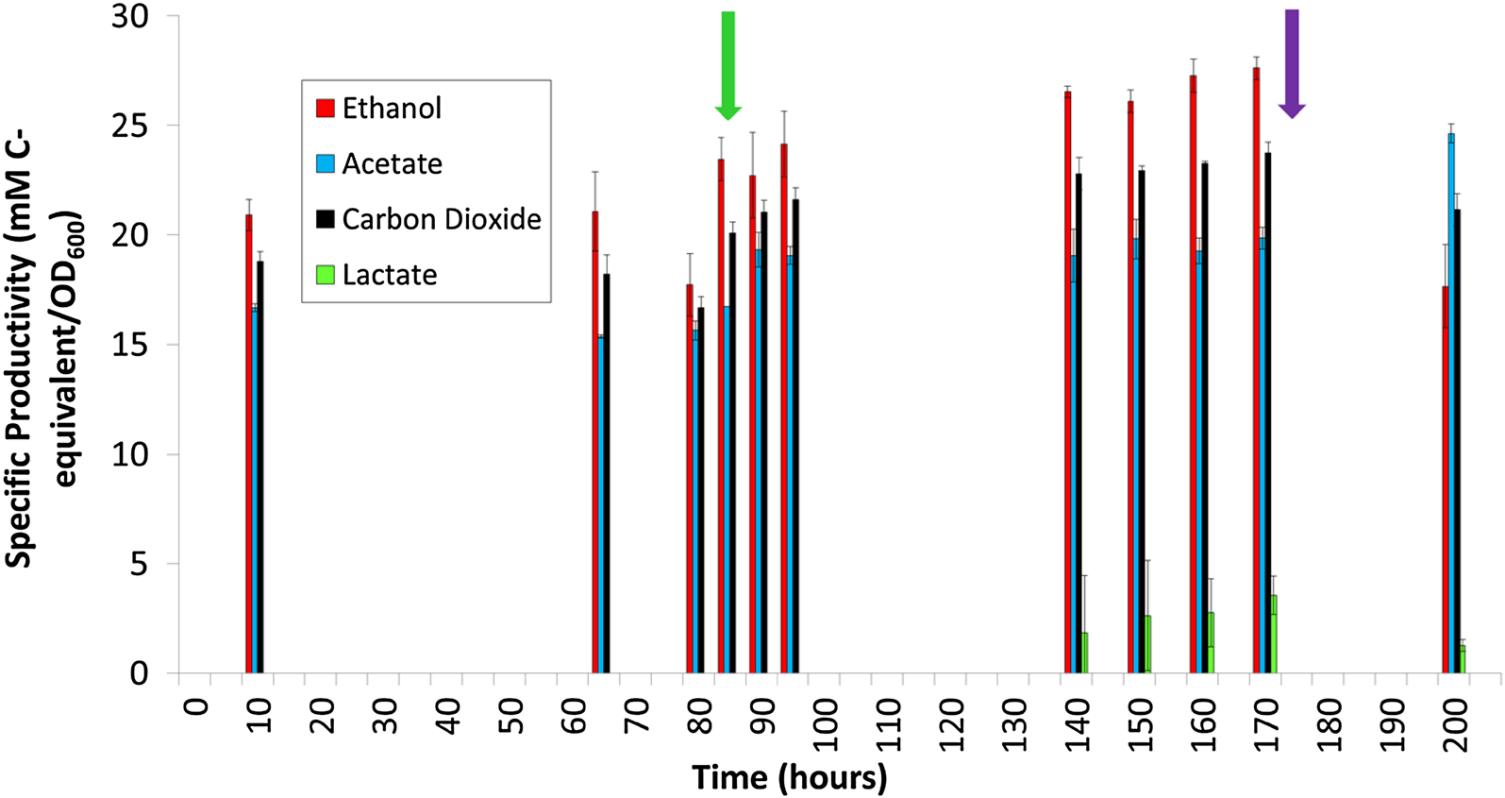
Fermentation productivity during addition of methyl viologen to chemostat culture. Specific fermentation products (converted to reflect the amount of carbon in the products; mol *C*-equivalents/L/OD_600_) are reported in equivalent carbon mole basis (e.g., 1 mol ethanol = 2 mol *C*-equivalent). Productivity in carbon moles is normalized to OD_600_ to account for changing cell yields observed across the time culture was exposed to methyl viologen. CO_2_ productivity is estimated by assuming one mole of CO_2_ is produced for each mole of ethanol and each mole of acetate produced.* Green arrow* indicates when methyl viologen exposure began and* purple arrow* indicates time when methyl viologen flushing from the reactor began

Carbon-limited (1.1 g/L cellobiose) chemostat grown cultures (0.1 h^−1^) were supplied with MTC medium containing methyl viologen (Fig. 1). Time “0” for methyl viologen addition was the point when the feed medium was switched from MTC medium to MTC medium containing methyl viologen (~88 h after the experiment began). Addition of methyl viologen to the reactor lowered cell density (as measured by fermenter OD_600_) (Fig. 1). An increase in specific ethanol production was observed concomitant with methyl viologen exposure, lower cell densities, and an approximate 50 mV decrease in redox potential (Figs. 1, 2). Throughout methyl viologen exposure, a total of 123 individual genes were significantly differentially expressed within at least one timepoint (defined as a log_2_ expression change of <−1.5 or >1.5 at a false discovery rate <0.05) (Table 1). After methyl viologen was flushed from the system, ethanol-specific productivity returned to pre-exposure levels (Fig. 2) and redox potential measurements returned to pre-exposure redox potential levels (Fig. 1a). Production of acetate remained unchanged before and during methyl viologen addition. Acetate concentration and yield increased after methyl viologen exposure was completed and as methyl viologen was being diluted out of the chemostats. CO_2_ and hydrogen production was not measured in the open chemostat system. Lactate began to be synthesized after 50 h of methyl viologen exposure, increasing a specific productivity from 1.85 to 3.55 mM/OD_600_. Before and throughout methyl viologen addition, all cellobiose substrate was utilized as no residual cellobiose or glucose was detected by HPLC analysis of samples from the fermenters. Specific fermentation products (converted to reflect the amount of carbon in the products; mol C-equivalents/L/OD_600_) increased throughout methyl viologen exposure. Ethanol and estimated CO_2_ (mol C-equivalents/L/OD_600_) increased 17.6 and 15.8 %, respectively, during 60 h of increasing methyl viologen exposure (Fig. 2). Increased alcohol concentrations have been observed after adding methyl viologen to cultures of *C.* *acetobutylicum,* which was attributed to decreased hydrogen production [28, 29]. This phenomenon was observed upon methyl viologen addition to *C.* *acetobutylicum* cultures and was attributed, in part, to increased transcription of butanol synthesis pathway genes [19]. In this study, transcription of ethanol synthesis genes was not increased significantly.

**Table 1:** Number of genes showing differential expression after beginning methyl viologen addition

	3 min	15 min	35 min	7 h	14 h	50 h	60 h
Number of genes up-regulated	0	14	1	3	1	21	36
Number of genes down-regulated	20	37	3	8	1	40	47

Significant differential expression was determined to be genes showing log_2_ fold change relative to untreated controls of >1.5 or <−1.5 at a 5 % false discovery rate. Significantly differentially expressed genes are highlighted in Additional file 1

Global transcriptional analysis suggests that two broad temporal expression patterns are present across genes that showed differential expression in at least one timepoint taken during methyl viologen exposure. Indeed, differential expression at 15 min was very similar to that sampled at 50 and 60 h. The first pattern observed was decreased expression at 15 min, followed by a return to pre-exposure expression, and finally decreased expression at 50 and 60 h. We interpret this result to suggest an expression response occurring immediately after initial exposure to methyl viologen (while concentrations of methyl viologen in the fermenters are low) followed by a decay of this response to pre-exposure conditions. Beginning at 50 h, expression levels return to levels similar to those seen at 15 min, but do so in a sustained fashion as those levels of expression are largely maintained for another 10 h. It should be mentioned that, by 50 h, concentrations of methyl viologen in the fermenters were estimated to be that of concentrations introduced in the feed carboy; 150 mg/L. We provide the entire significant gene list and focus below on predominant and possibly coordinated systems.

### Decreased transcription of redox-active pathways

Methyl viologen can occur in three reduction states, MV^0^, MV^+^, and MV^2+^, with the redox potential of the most reduced form being similar to that of electron transfer proteins, flavodoxins and ferredoxins [30, 31]. Methyl viologen can be used in place of natural electron acceptors and oxidoreductase proteins in nitrogenase [32], nitrate reductase [33], and other enzyme assays. In addition to these known enzymatic interactions, methyl viologen has also been shown to interact directly with iron-sulfur clusters and, in some cases, degrade them [34]. Fe-S cluster degradation by methyl viologen and other redox-active compounds has been shown to initiate wide-ranging transcriptional changes through induction of the SoxRS transcription factor system [35]. Using mechanisms similar to these, it is conceivable that methyl viologen can interact with redox metabolism indirectly in *C. thermocellum* and, as such, our experimental system may be capturing transcriptional outcomes of these mechanisms in addition to direct interactions facilitated by enzymes and redox chemistry with redox-active intracellular metabolites.

**Fig. 3:**
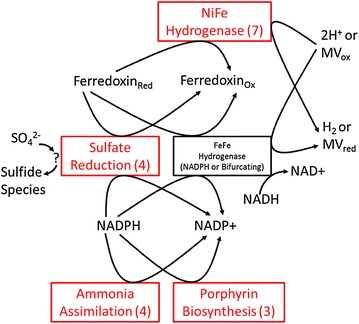
Global view of main of *C. thermocellum* DSM 1313 transcriptional responses to methyl viologen. *Red* indicates decreased transcription of genes in the indicated systems. *Numbers in parenthesis* are the number of genes in the indicated pathways that show decreased expression during methyl viologen addition

Four redox-active pathways showed decreased transcription at various timepoints after methyl viologen addition; sulfur transport and assimilation, ATP-dependent GS-GOGAT ammonia assimilation, porphyrin and siroheme biosynthesis, and the [NiFe] Fd-dependent hydrogenase (Figs. 1, 2, 3; Additional file 2). All four systems show decreased transcription at 50 h (3000 min)–60 h (3600 min) after methyl viologen addition when estimated methyl viologen concentration in the reactor is greatest and the fermenter redox potential was the lowest. GS-GOGAT and some genes of the [NiFe] system and gene cluster show decreased transcription at three and 15 min after beginning methyl viologen addition.

### Ammonia assimilation

Previous studies have shown that the malic enzyme is allosterically activated by ammonia for the conversion of malate to pyruvate and it is likely the primary carbon flux channel under certain growth conditions in *C. thermocellum* [36]. The putative DSM 1313 malic enzyme gene (Clo1313_1878) showed slightly greater transcript levels after methyl viologen treatment, but not at levels considered significant (Additional file 2). Intracellular metabolites, such as ammonia, and enzymatic activities were not measured as part of present study. We observed genes encoding the GS-GOGAT system showed decreased transcription (Table 2). Both glutamine synthetase (GS) and glutamate synthase (GOGAT) showed decreased expression (log_2_ differential expression of −1.3 to −2.4 relative to untreated controls) at 3 min and 15 min and also at 3000 and 3600 min [log_2_ differential expression of −0.6 to −1.5 relative to untreated controls (Table 2)]. Other genes annotated as glutamine synthetase (Clo1313_2038, Clo1313_2031, and Clo1313_1357) in KEGG and predicted to catalyze the same enzymatic reaction (E.C. 6.3.1.2) showed no differential transcription in this study. One other gene annotated as a glutamate synthase (Clo1313_1849) also did not show any differential transcription in this study. Recent reports suggest that Clo1313_1849 actually encodes the NfnA subunit of NfnAB [13].

**Table 2:** Differential expression for GS-GOGAT ammonia and [NiFe] hydrogenase genes

Locus tag	Gene product	Metabolic function	${ \log }_{ 2} \left( {\frac{\text{Expression (Time Exposed)}}{\text{Expression (Unexposed)}}} \right)$						
			3 min	15 min	35 min	7 h	14 h	50 h	60 h
Clo1313_2303	Glutamine synthetase catalytic region	GS-GOGAT Ammonia fixation	*−2.2*	*−2.4*	*−0.9*	*1.0*	*1.2*	*−1.5*	*−1.3*
Clo1313_2036	Glutamine amidotransferase class-II		*−1.3*	*−2.1*	−0.4	*1.6*	*1.5*	−0.6	−0.7
Clo1313_2035	Ferredoxin-dependent glutamate synthase		*−2.0*	*−2.2*	*−0.6*	*1.2*	*1.2*	*−1.1*	*−0.9*
Clo1313_2034	4Fe-4S ferredoxin iron-sulfur binding domain-containing protein		*−2.3*	*−2.4*	*−0.9*	*0.7*	*0.7*	*−1.5*	*−1.3*
Clo1313_0564	Hydrogenase expression/formation protein HypE	NiFe hydrogenase	*−0.4*	*−1.4*	*−0.5*	*−0.9*	*−0.7*	*−1.3*	*−1.7*
Clo1313_0565	Hydrogenase expression/formation protein HypD		*−0.4*	*−0.6*	−0.1	*−0.3*	*−0.3*	*−0.7*	*−0.9*
Clo1313_0566	Hydrogenase assembly chaperone hypC/hupF		*−0.5*	*−0.8*	−0.2	*−0.4*	*−0.3*	*−0.9*	*−1.2*
Clo1313_0567	(NiFe) Hydrogenase maturation protein HypF		*−0.7*	*−0.9*	0.0	*−0.5*	−0.3	*−1.0*	*−1.0*
Clo1313_0568	Hydrogenase accessory protein HypB		−0.1	*−0.5*	*0.5*	0.0	0.0	*−0.4*	*−0.9*
Clo1313_0569	Hydrogenase expression/synthesis HypA		*−0.3*	*−0.9*	0.1	*−0.3*	−0.2	*−0.7*	*−1.1*
Clo1313_0570	4Fe-4S Ferredoxin iron-sulfur binding domain-containing protein		*−0.4*	*−1.2*	0.2	−0.2	−0.2	*−0.8*	*−1.3*

Values in italics indicate statistical significance using a 5 % false discovery rate

Clo1313_2036 and Clo1313_2035, the genes putatively encoding two subunits of glutamate synthase, are part of a gene cluster (Clo1313_2030–Clo1313_2036) that showed similar expression behavior (Additional file 2). Clo1313_2034 is annotated as a 4Fe-4S ferredoxin iron-sulfur binding domain-containing protein. Top global protein-BLAST similarity scores are to iron-sulfur cluster containing ferredoxin in other strains of *C. thermocellum* and other *Clostridia*. Both the GS-GOGAT system and glutamate dehydrogenase are annotated as being NADPH dependent, and to the best of our knowledge, ferredoxin-dependent GS-GOGAT activity has not been assayed for in *C. thermocellum* and NADH-dependent GOGAT activity was not found in crude cell lysates of *C. thermocellum* DSM 1273 [37]. Glutamate synthesis from glutamine using glutamate synthase may compete for reductant with other redox processes, including the reduction of methyl viologen. Glutamine biosynthesis also requires glutamate, ATP, and ammonium. Reduced expression of the GS-GOGAT system may be the result of added demand for reductant and/or altered cofactor pool states introduced by the presence of oxidized methyl viologen, may reflect lower intracellular ATP levels and/or reflect less cellular demand for glutamine. Such altered states may trigger a transcriptional response towards preserving reductant and/or ATP at the expense of glutamine and glutamate production using this GS-GOGAT system.

Transcriptional regulation of ammonia transport, the GS-GOGAT system, and glutamate dehydrogenase appears to be complex, multi-layered, and varies greatly between organisms [38, 39]. A transcriptional regulator of ammonia assimilation characterized in *C.* *acetobutylicum*, relies on expression of a *nitR* antiterminator protein and an antisense RNA [40]. Clo1313_2030 is annotated as a response regulator receiver and ANTAR domain protein. The Clo1313_2030 gene showed decreased expression mirroring that of the gene cluster containing the genes Clo1313_2030 through Clo1313_2036 and its product may provide an antiterminator role sensitive to ammonia concentrations similar to the one described in *C.* *acetobutylicum*. The AmtB and PII proteins contribute to ammonia transport and regulation in different systems [38]. Clo1313_2260 contains putative AmtB and PII domains that are 51 and 81 % similar to *C.* *acetobutylicum* ATCC 824 CA_C0682 and CA_C0681 at the protein level, respectively. In this study, Clo1313_2260 was not significantly differentially expressed.

Glutamate dehydrogenase (Clo1313_1847) catalyzes an alternative mechanism for synthesizing glutamate and assimilating ammonia and glutamate dehydrogenase (Clo1313_1847) was not differentially expressed in this study. Glutamate dehydrogenase activity was shown to be much higher than GOGAT in *C. thermocellum* DSM 1273 extracts, suggesting it is the predominant method for assimilating ammonia and generating glutamate [37]. However, these experiments were conducted at a maximum nitrogen concentration of 18 mM, supplied as ammonia and 0.2 % yeast extract. MTC medium used in this experiment contained more inorganic nitrogen in the form of both urea (33.3 mM) and ammonia (as ammonium chloride at 28 mM). The medium used in this experiment and routinely for *C. thermocellum* growth contains a large excess of nitrogen source and it is possible to culture *C. thermocellum* in medium containing less nitrogen [41].

Glutamate is a precursor to many biosynthetic pathways, including the biosynthesis of porphyrin rings. Porphyrin ring biosynthesis also showed decreased transcription upon exposure to methyl viologen. Decreased demand for glutamate or increased availability may have led to decreased transcription of GS-GOGAT. Other putative KEGG pathways using glutamate as a precursor did not show differential expression. By contrast, increased transcription and translation of both glutamine synthetases (Cthe_0196, Cthe_1539) were observed after *C.* *themocellum* ethanol stress, which may have been related to metabolite shuttling into carbamoyl-P, a precursor to pyrimidine, arginine and proline biosynthesis [16], or possibly glutamine synthetase and the ammonia assimilation pathways indirectly assist in reoxidizing redox cofactors.

#### Sulfate transport and metabolism

*Clostridium thermocellum* strain ATCC 27405 has genes for and can assimilate sulfate [41]. As in strain ATCC 27405, sulfate transport genes (Clo1313_0114– Clo1313_0117) and putative assimilatory sulfate reduction genes (Clo1313_0119, Clo1313_0120, and Clo1313_0124) are co-located on the DSM 1313 chromosome. In this study, these genes show similar expression patterns and lower expression levels under methyl viologen stress with log_2_ differential expression of −0.9 to −1.6 relative to untreated controls at 3000 and 3600 min (Table 3), which is consistent with methyl viologen-exposed *C. acetobutylicum* cells [19] and indicates potential similarity in the physiological and regulatory responses between these organisms. Adjacent to sulfur-related genes, the *C.* *thermocellum* Clo1313_0107 gene encodes a putative transcriptional regulator IscR [14], and its expression increased following methyl viologen exposure (Table 3). Differential expression profiles for Clo1313_0107 (*iscR*) and porphyrin biosynthesis genes are similar when exposed to furfural or heat [14]. Further studies to generate and characterize a Clo1313_0107 deletion strain are required to elucidate its possible roles in sulfate uptake and metabolism, stress responses, and gene regulatory networks. Because methyl viologen is a potential alternative electron sink to reoxidize reduced intracellular cofactors, *C. thermocellum* DSM 1313 could potentially repress genes involved in sulfate transport and reduction when exposed to methyl viologen in response to a decreased need for electron acceptors. Sulfate reduction is also ATP dependent and it may benefit *C. thermocellum* DSM 1313 energetically to decrease expression and/or activity of this pathway.

**Table 3:** Differential expression information for genes Clo1313_0107 through Clo1313_0124

Locus tag	Gene product	Metabolic function	${ \log }_{ 2} \left( {\frac{\text{Expression (Time Exposed)}}{\text{Expression (Unexposed)}}} \right)$						
			3 min	15 min	35 min	7 h	14 h	50 h	60 h
Clo1313_0107	Transcriptional regulator, Rrf2 family	Putative IscR transcription factor	*1.1*	0.2	*2.4*	*2.1*	*1.9*	*1.3*	*1.7*
Clo1313_0109	Precorrin-6X reductase	Porphyrin Biosynthesis	*−0.5*	*−0.4*	*0.2*	−0.2	−0.1	*−0.7*	*−1.2*
Clo1313_0112	Delta-aminolevulinic acid dehydratase		*−0.8*	−0.5	−0.1	*−0.5*	−0.3	*−1.2*	*−1.5*
Clo1313_0113	Glutamate-1-semialdehyde-2,1-aminomutase		*−0.9*	−0.6	0.1	−0.3	−0.3	*−1.2*	*−1.6*
Clo1313_0115	Sulfate ABC transporter, inner membrane subunit CysT	Sulfate ABC transporter	*−0.6*	−0.1	*0.5*	*0.4*	*0.4*	*−1.2*	*−1.3*
Clo1313_0116	Sulfate ABC transporter, inner membrane subunit CysW		*−0.6*	0.1	*0.5*	0.4	0.4	*−1.0*	*−1.4*
Clo1313_0118	Adenylylsulfate reductase, thioredoxin dependent	Sulfate reduction	*−0.9*	−0.4	*0.5*	0.1	0.1	*−1.4*	*−1.6*
Clo1313_0124	Nitrite and sulfite reductase 4Fe-4S region		*−1.4*	−0.2	0.2	0	−0.1	*−0.9*	*−1.5*

Values in italics indicate statistical significance using a 5 % false discovery rate

#### Porphyrin biosynthesis

Many redox-active enzymes are iron-sulfur containing proteins and siroheme is often a necessary redox-active cofactor. The genes Clo1313_0108–Clo1313_0113 putatively encode for proteins involved in porphyrin/siroheme biosynthesis and they are adjacent to genes involved in sulfate transport/reduction as well as genes potentially involved in regulation. The Clo1313_0109, Clo1313_0112, and Clo1313_0113 genes show differential expression at 3000 min after methyl viologen addition (log_2_ differential expression of −0.7, −1.2, and −1.2, respectively, relative to untreated controls), and show the largest differential expression after 3600 min (log_2_ differential expression of −1.2, −1.5, and −1.6, respectively, relative to untreated controls), when estimated methyl viologen concentration is the highest (Table 3). Three other putative porphyrin biosynthesis genes in this cluster do not meet criteria for significant differential expression. Clo1313_0124 is annotated as nitrite and sulfite reductase, predicted to contain an iron-sulfur/siroheme binding site, and also shows decreased transcription under methyl viologen exposure. Clo1313_0124 is annotated to be ferredoxin dependent. Expression of Clo1313_0109 was shown to increase upon exposure to *Populus* hydrolysate [17] and decreased expression 10, 30, and 60 min following exposure to increased ethanol concentration [16], which suggests, along with results from this study, that transcription of genes responsible for porphyrin biosynthesis is sensitive to cellular redox potential and/or the redox potential of the fermentation environment. With methyl viologen potentially providing an extra sink for cellular reductant through a process mediated by hydrogenases or occurring directly, there may be less overall demand for these proteins mediating redox reactions. Unbound heme and some of the metabolic intermediates of the tetrapyrrole biosynthesis pathway are known to be cytotoxic and can be recycled [42]. *Pseudomonas aeruginosa* appears to regulate early stages of heme biosynthesis in accordance with growth state and overall need for heme enzyme cofactors [43]. Many microorganisms have been shown to limit the amount of accumulated 5-aminolenulevulenate [42], suggesting it may be particularly cytotoxic and/or the precursor to a rate-limiting step in tetrapyrrole biosynthesis. Thus, it may be advantageous for *C. thermocellum* DSM 1313 to regulate this pathway and Clo1313_0107 may play a role.

#### [NiFe] hydrogenases

Methyl viologen has been shown to mediate re-oxidation of reduced cellular species through interaction with hydrogenase proteins [44, 45]. *C. thermocellum* DSM 1313 encodes four hydrogenases; three [FeFe] hydrogenases and one [NiFe] hydrogenase. No differential expression was observed in genes encoding any of the three [FeFe] hydrogenase systems in response to methyl viologen exposure. It is thought that two of the three [FeFe] hydrogenases are bifurcating hydrogenases [46], necessarily requiring one mole of NADH and one mole of reduced ferredoxin to produce one mole of hydrogen. The third [FeFe] hydrogenase is thought to be NADPH dependent. It has been shown that the NADPH-dependent hydrogenase activity was greater than either reduced ferredoxin or NADH-dependent hydrogenase activity [47, 48]. Further, low amounts of Ech hydrogenase protein was quantified [46] and poor transcription of *ech* genes [49] was found using reverse transcriptase PCR from *C. thermocellum*. In contrast, we observe all genes in the *ech* hydrogenase gene cluster to be highly transcribed in the conditions used in this study, based on LOWESS normalized hybridization intensity. All but one of the genes encoding *ech,* an [NiFe]-containing hydrogenase (Clo1313_0564–Clo1313_0570) show transient initial decreased transcription at 3 and 15 min (log_2_ differential expression −0.3 to −1.2, relative to untreated controls) after beginning methyl viologen exposure, followed by a return to unchanged levels of transcription (significant log_2_ differential expression not less than −0.4 or greater than 0.2, relative to untreated controls). Expression of this gene cluster again decreased at 3000 and 3600 min (log_2_ differential expression −0.7 to −1.3, relative to untreated controls) after beginning methyl viologen exposure when the concentration of methyl viologen in the reactor is highest. All genes in this cluster appear to observe this temporal expression behavior (Table 3). It would be interesting to determine whether or not the [NiFe] hydrogenase is ferredoxin dependent and biochemically characterize its relative contribution to hydrogen metabolism to better understand co-factor re-oxidation capacity and dynamics in *C. thermocellum*.

*Clostridium thermocellum* DSM 1313 has been reported to have overflow metabolic pathways, potentially related to overly reduced intracellular conditions and a need to reoxidize redox cofactors [9, 11]. [NiFe] *ech* hydrogenase transcription does not appear to be transcriptionally linked to ATP generation or establishing/modulating the proton motive force in *C. thermocellum*, as there was no significant differential expression of the ATP synthase genes (Clo1313_2935–Clo1313_2942) observed during addition of methyl viologen to the culture. In different systems [NiFe], hydrogenase is known to be controlled by a number of different mechanisms and responsive to many different environmental and physiological cues, such as H_2_, O_2_, and CO as well as the FnrT transcription factor [50, 51]. [NiFe] hydrogenases have also been linked to nitrogenase activity through electron transfer to membrane-bound Rnf [50]. *C.* *thermocellum* encodes genes with putative functions for N_2_ fixation (Clo1313_2331, Clo1313_2332, and Clo1313_2339). Clo1313_2331 and Clo1313_2339 show significant decreased transcription at 3 min after methyl viologen addition and differential expression in an equivalent *C. thermocellum* ATCC 27405 gene, Cthe_1573 *(nifH)* showed increased expression 240 min after exogenous ethanol addition to batch culture [16]. *C.* *thermocellum* DSM 1237 was reported to show nitrogen-fixing activity in vitro, via reduction of acetylene, though doubt was cast as the activity was not seen to be repressed by added ammonia [37]. *C. thermocellum* diazotrophic growth was tested and under the conditions assayed nitrogen fixation was not detected [41]. *C.thermocellum* electron transfer requires greater study.

### Transcription differences in other systems

#### Transporters

Eight annotated transporters found to be significantly differentially expressed during at least one timepoint under methyl viologen treatment. A CorA family transporter annotated as a magnesium transporter showed relatively strong increased transcription at 3000 and 3600 min after methyl viologen addition. Five transporters annotated as being ATP binding showed decreased transcription at least one timepoint during methyl viologen exposure.

#### Sporulation

*Clostridium thermocellum* strain ATCC 27405 sporulates at low frequencies (maximum ~7 %) and not under certain conditions such as in response to low carbon or nitrogen environments [52], which confounds global population-based analyses. In this study, three sporulation genes showed significant decreased transcription (false discovery rate <0.05 and log_2_ differential expression less than −1.5 relative to untreated controls); Cthe_3070, Cthe_0044, and Cthe_2948. The initial sporulation developmental stages transcription factor, *spoOA* (Clo1313_1409), showed significant changes in expression at 3 and 15 min after beginning methyl viologen addition (log_2_ differential expression of 0.4 and −0.8, respectively). Two of the four histidine kinases (Clo1313_0286 and Clo1313_1973) previously identified as agents of sporulation control [53] showed increased differential transcription at 3000 (log_2_ 1.1 and 1.1, respectively) and 3600 min (log_2_ 1.0 and 1.5, respectively) after beginning methyl viologen exposure. Clo1313_1973 also showed relatively strong decreased transcription at 3 min (significant log_2_ differential expression of −1.5). In addition to spore formation, *C.* *thermocellum* (strain ATCC 27405) can develop an L-form morphology which is another non-growth cell state characterized by low metabolic activity with a spherical or pleomorphic morphology [52]. Spores and L-form cells were not observed together in a previous study [52]. Much remains to be elucidated regarding signal-transduction and regulatory cascades controlling these processes.

#### Transposases and phage-associated gene expression

Two genes annotated as transposases (Clo1313_0662 and Clo1313_2686) showed increased transcription after 3600 min (significant log_2_ differential expression of 1.8 and 1.7, respectively). These two genes appear to be highly transcribed before and during exposure to methyl viologen (LOWESS normalized expression intensity ranged from log_2_ 11.4–13.4). Transposons have been shown to interrupt and inactivate the *C. thermocellumcipA* genes [54]. *C. thermocellum* ATCC 27405 transcriptomic studies have shown differential expression for transposases under different growth and stress conditions [14, 16]. *C. thermocellum* strains differ in their phage and CRISPR content [55]. Increased expression of genes associated with a putative phage island (Clo1313_2379–Clo1313_2409) was observed in one of two replicate samples taken after exposure to methyl viologen and immediately prior to beginning exposure to H_2_O_2_, after 3600 min (60 h) of re-equilibration growth on MTC not amended with either stressor chemical (Additional file 2). The majority of genes from this phage island were found to show significant increased transcription in *C.* *thermocellum* ATCC 27405 after 30, 60, and 120 min of exposure to furfural and ethanol, with the largest transcriptional increases coming 30 min after exposure and dropping gradually at 60 and 120 min for both conditions [14, 16]. This gene region *C.* *thermocellum* DSM 1313 does not contain ‘att sites’ characteristic of functional lysogenic phage, though the closely related *C. thermocellum* ATCC 27405 does [55]. Genes for phosphate transport and regulation are adjacent to recombinase and phage genes in *C. thermocellum* ATCC 27405, which indicates possible mechanisms for horizontal gene transfer [14]. Observed strain genome differences and differential expression from this and prior studies suggest transposons, bacteriophage, and CRISPR systems may have and may continue to play important roles in *C.* *thermocellum* evolution, although the functionality and implications for these systems have only begun to be investigated.

### Hydrogen peroxide addition to chemostat culture

Compared to untreated fermentation redox potential (approximately −500 mV), fermenter redox sharply and briefly increased by approximately 100 mV initially and then remained approximately 25 mV higher than the control during prolonged H_2_O_2_ exposure. However, no genes showed significant differential expression (log_2_ fold change >1.5 or <−1.5 and a 5 % false discovery rate) after chemostat hydrogen peroxide exposure and only a few gene comparisons showed significant differences (below fold threshold) after 3 min of H_2_O_2_ exposure. There was no apparent change in acetate, ethanol, lactate, or estimated CO_2_ cell yields (moles C-equivalent/OD_600_). Furthermore, addition of H_2_O_2_ under the conditions mentioned previously had no prominent effect on fermenter OD_600_. Results are shown in Additional file 3.

It is likely the concentration of hydrogen peroxide introduced into the fermenter, though enough to alter the fermentation redox potential was insufficient to induce detectable transcriptional-level changes. The chemostat cultures were metabolically active and in greater densities compared to the preliminary batch screening assays where hydrogen peroxide was added to lag-phase cultures, which may explain the poor transcriptional response for this condition. Redox couples within the cell, such as the GSH/GSSG couple [56], are able to act as redox buffers and provide cells protection against unfavorable environmental redox conditions while not necessarily requiring changes in transcription. It is conceivable that *C. thermocellum* could have used similar systems to modulate the hydrogen peroxide induced redox perturbation it was exposed to in this experiment, instead of systems requiring changes to its transcription profile. Additionally, hydrogen peroxide is a susceptible to degradation and chemical change under the conditions it was used in this experiment. A portion of the hydrogen peroxide may have degraded in the process steps leading up to its introduction into the fermenters. Though we recognize this possibility, we infer a change in redox potential after the addition of of hydrogen peroxide to the fermenters as evidence that the hydrogen peroxide treatment did have an oxidizing effect on the culture, though possibly not as large as anticipated.

## Conclusions

We examined *C. thermocellum* DSM 1313 redox metabolism by analyzing the transcriptional response to gradual addition of methyl viologen to steady-state cultures. Specific ethanol productivity increased steadily during methyl viologen addition, likely due to altered redox state, while OD_600_ dropped initially after methyl viologen addition and then recovered slightly and stabilized after 3000 min exposure. The redox potential of the fermentation was stable at −500 mV before methyl viologen addition and began dropping immediately after methyl viologen addition began and stabilized at −550 mV after approximately 100 min. We observed a number of redox-active and ATP-requiring systems showing decreases in transcription as methyl viologen was added to chemostat cultures. Genes encoding sulfate transport and reduction, glutamate synthase-glutamine synthetases (the GS-GOGAT system), and portions of porphyrin biosynthesis showed differential expression in response to added methyl viologen, suggesting their involvement in mediating *C. thermocellum* redox homeostasis and energy metabolism. Genes encoding subunits and accessory proteins of the sole [NiFe] hydrogenase differentially expressed while those of [FeFe] hydrogenases did not. Other genes involved in transport, sporulation, and transposons also showed differential expression upon exposure to methyl viologen. This global profiling study provides gene targets for future studies to elucidate the relative contributions of prospective pathways for co-factor pool re-oxidation and *C.* *thermocellum* redox homeostasis.

## Methods

### Strains, media, and materials

*Clostridium thermocellum* DSM 1313 was obtained from the German Collection of Microorganisms and Cell Cultures (DSMZ) and grown in MTC medium essentially described previously [57]. Briefly, MTC was prepared as a mixture of five solutions and contained the following (final concentrations): Solution A: 1.1 g/L cellobiose, 2 mg/L resazurin. Solution B: 2.12 g/L C_6_H_7_K_3_O_8_, 1.25 g/L C_6_H_8_O_7_-H_2_0, 1 g/L Na_2_SO_4_, 1 g/L KH_2_PO_4_, 2.5 g/L NaHCO_3_. Solution C: 1.5 g/L NH_4_Cl, 2 g/L CH_4_N_2_O. Solution D: 1 g/L MgCl_2_-6H_2_0, 0.2 g/L CaCl_2_-2H_2_0, 0.1 g/L FeCl_2_-2H_2_O, 1 g/L C_3_H_7_NO_2_S-HCl-H_2_O. Solution E: 20 mg/L Pyridoxamine dihydrochloride, 4 mg/L P-aminobenzoic acid, 2 mg/L D-biotin, 2 mg/L vitamin B12. Solution F: 0.5 mg/L MnCl_2_-4H_2_O, 0.5 mg/L CoCl_2_-6H_2_O, 0.2 mg/L ZnCl_2_, 0.05 mg/L CuCl_2_-2H_2_O, 0.05 mg/L H_3_BO_3_, 0.05 mg/L Na_2_MoO_4_-2H_2_O, 0.05 mg/L NiCl_2_-6H_2_O. Media prepared for bottle-based batch fermentations contained 5 g/L MOPS sodium salt. Media for chemostat cultivation did not contain MOPS sodium salt. All media were made anaerobic by sparging with N_2_ gas.

### Preliminary batch fermentations

*Clostridium thermocellum* DSM 1313 was grown in MTC medium containing 1.1 g/L cellobiose. Cultures were inoculated into hungate tubes containing 10 mL of medium and initial headspace of 10 % CO_2_ (v/v), 5 % H_2_ (v/v), and the balance N_2_. Methyl viologen was added to medium which was then pre-warmed overnight prior to inoculation. H_2_O_2_ was added to pre-warmed medium immediately prior to inoculation. Cultures were inoculated with 1 mL of overnight grown culture and growth was monitored using a Milton Roy Spectronic 21D UV–Visible Spectrophotometer (Milton Roy Company). Soluble fermentation products were measured using HPLC (see HPLC analysis portion of methods) and headspace H_2_ % was measured using an Agilent 6850 GC equipped with a thermal conductivity detector (TCD) for CO_2_ and H_2_ quantification (Agilent Technologies, USA).

In preliminary batch fermentations, the end of fermentation was determined based on a decrease in culture OD_600_, common in *C. thermocellum* batch fermentations and thought to correspond with the onset of stationary phase. Samples for end-product determinations were collected immediately after the final OD600 reading was taken. End-products were normalized to the average maximum OD600 achieved during batch fermentation.

### Chemostat growth and stress application

*Clostridium thermocellum* DSM 1313 was grown at 55 °C in duplicate 1 L (total vessel capacity 1.3 L) chemostat cultures using water-jacketed BioFlo110 bioreactors (New Brunswick Scientific, Edison, NJ, USA) with 1.1 g/L cellobiose as the carbon source in MTC medium, which was fed at a dilution rate of 0.1 h^−1^. Temperature, pH, and agitation speed were monitored and controlled during fermentation. Culture pH was monitored using pH electrodes (Mettler-Toledo, Columbus, OH, USA) and the pH control set point was maintained at 7.0 by automatic titration with 3 N KOH. Fermenter redox potential was measured using DPAS K8S 325 combination redox probes (Mettler-Toledo) attached to an independent signal transducer and readout (Sartorious Stedim Biotech, model # 8890354). Redox probes were checked for accuracy using new redox probe calibration solutions (Ricca Chemical Company catalog #4330-16, Ricca Chemical Company catalog #9880-16, Orion Application Solution # 967901). Agitation was supplied by a central impeller with two paddled rotors maintained at 200 rpm and no additional baffles. Culture turbidity was measured taking optical density readings at 600 nm using a Genesys 20 spectrophotometer (Thermo Fisher Scientific Inc., Waltham, MA, USA).

Fermenters were inoculated using 100 mL of overnight cultures into 900 mL of neutral MTC previously sparged overnight with N_2_ gas. After inoculation, N_2_ sparging was stopped and for the duration of chemostat growth N_2_ was flushed into the fermenter headspace. Chemostat steady-state growth was defined as being at least 50 h of continuous growth during which the culture OD_600_ fluctuated less than 5 %. Individual chemical stressors were added to medium feed carboys at final concentrations of 10 mg/L H_2_O_2_ or 150 mg/L methyl viologen and fed into the reactors with medium feed over 60 h (6 retention times). Chemostat methyl viologen and H_2_O_2_ concentrations were selected based on preliminary batch experiments (Additional files 1, 3, respectively) and in each case, culture growth rates were not impacted by greater than 50 % and final fermentation product concentrations (normalized to maximum OD_600_) changed by >20 %. Upon completion of each chemostat stress treatment, a 500 mL volume was withdrawn from each vessel and then reactors were re-filled in fed-batch mode to 1 L with only MTC medium and then chemostat operation was resumed. Remaining stressor was allowed to wash out for another 50 h (5 retention times) and a re-equilibration period of 50 h followed before the next stress application. Methyl viologen and hydrogen peroxide concentrations or chemical stability were not measured or assayed directly in the fermenters or feed carboys. Redox potential was measured and recorded in the fermenters to ensure stress chemical additions were changing the fermentation redox potential and the overall redox environment. Chemostat culture integrity was checked periodically by microscopy and PCR amplification and sequencing of the 16S rRNA gene using forward sequencing primer AGAGTTTGATCCTGGCTCAG and reverse sequencing primer GGGCGGTGTGTACAAGG. Biofilm growth or excessive frothing was not observed or mitigated during chemostat growth.

### Fermentation product analysis using high-performance liquid chromatography (HPLC)

HPLC samples were collected by centrifugation at 13,000 rpm in a microcentrifuge and passed through a 0.22 µm filter, acidified with 11.6 mN H_2_SO_4_ and stored at 4 °C until analyzed using a LaChrom Elite System (Hitachi High Technologies America, Inc., CA, USA) fitted with a Aminex HPX 87H HPLC column (300 × 7.8 mm) (Bio-Rad, Hercules, Dallas, TX, USA) kept at 60 °C and using a mobile phase of 5 mM H_2_SO_4_ with a flow rate of 0.5 mL/min for 35 min per sample. Eluted compounds were detected by a refractive index detector (Model L-2490) and quantified via retention time and peak areas. Standard curves were used to quantify peak areas from samples and each sample was injected at least twice.

### RNA isolation, cDNA synthesis, microarray hybridization, and data analysis

Briefly, 50 mL aliquots from chemostat cultures were harvested by centrifugation (8000 rpm, 4 °C, 4 min using a Sorvall RC5C plus centrifuge), the supernatant decanted and removed and the remaining pellets were quickly frozen in liquid nitrogen and stored at −80 °C. Frozen cell pellets were resuspended in TRIzol reagent (Invitrogen, Carlsbad, CA, USA), lysed by adding 1.5 mL of cell pellet/TRIzol mixture to 2 mL screw top tubes containing 800 mg of ashed glass beads (#11079101, Biospec products, Bartlesville, OK, USA) followed by bead beating for three 20 s cycles at 6500 rpm using a Precellys-24 (Bertin Technologies, Montigny-le-Bretonneux, France). Total cellular RNA was purified using a QIAGEN RNeasy Mini kit according to the manufacturer’s instructions, which included an on column RNase-free DNase treatment to digest residual chromosomal DNA. RNA was quantified using a NanoDrop ND-1000 spectrophotometer (NanoDrop Technologies, DE, USA) and a Bioanalyzer 2100 (Agilent Technologies Inc., CA, USA). Double-strand (ds) cDNA was generated from purified RNA using a ds-cDNA synthesis kit (Invitrogen Life Technologies, NY, USA), which was subsequently labeled, hybridized, and washed according to the NimbleGen (Roche NimbleGen, IN, USA) protocols as described previously [16]. *C.* *thermocellum* transcriptome profiles were generated using an established *C. thermocellum* strain 27405 DNA microarray platform that contains 5–7 unique probes per gene and with three technical replicates for each unique probe, as described previously [14–16]. *C. thermocellum* strains DSM1313 and ATCC27045 are closely related; with average nucleotide identities (ANIs) of 99.6 and 99.3 % in reciprocal genome comparisons, indicating the strains are very closely related. Strain ATCC 27405 has a putative high-affinity phosphate transport system that DSM1313 lacks [16] and ATCC 27405 contains additional prophage and restriction-modification sequences [55]; hence the ATCC27405 DNA microarray was suitable to assess *C. thermocellum* strain DSM1313. Hybridizations were conducted using a 12-bay hybridization station (BioMicro Systems, Inc., UT, USA). Microarrays were dried and then scanned with a Surescan high-resolution DNA microarray scanner (5 μm) (Agilent Technologies, CA, USA), and the images were quantified using NimbleScan software (Roche NimbleGen). Raw data was log_2_ transformed and imported into the statistical analysis software JMP Genomics 6.0 software (SAS Institute, NC, USA). Data were normalized together using a single round of the LOWESS normalization algorithm within JMP Genomics. Distribution analyses were conducted before and after normalization as a quality control step. An ANOVA was performed in JMP Genomics to determine differential expression levels between untreated, equilibrated conditions immediately prior to stressor exposure and timepoints during stressor exposure using the false discovery rate (FDR) testing method (*p* < 0.05) and array slide used as a random variable. Microarray data have been deposited in the NCBI GEO database [GSE71465]. The Pathway Tools software [58] was used to overlay differential expression data onto a *C. thermocellum* metabolic map. The ‘Omics Viewer’ function within the software was used along with the curated genome-inferred metabolic model of *C. thermocellum* as provided in the Pathway Tools Pathway/Genome Database. Log2 normalized differential transcription as computed by differential expression analysis done using JMP genomics was imported and used to overlay the data.

### Microarray validation using real-time quantitative-PCR (RT-qPCR)

Microarray data were validated using RT-qPCR, as described previously [16]. Oligonucleotide sequences of the primers targeting five genes are listed in Additional file 4. Correlations between differential expression values obtained by RT-qPCR analysis and microarray analysis gave an R^2^ value of 0.94 indicating expression values obtained by microarray analysis are of good quality.

### Methyl viologen incubation experiment

*Clostridium thermocellum* DSM 1313 cells prepared under different conditions (washed and resuspended in distilled water, actively growing in medium, stationary phase in medium, autoclave killed, grown with and without resazurin) were incubated in 150 mg/L methyl viologen (Sigma Aldrich) to determine the origin of the reductant being used to reduce methyl viologen by growing cultures. To prepare live cell aliquots containing medium, 1 mL of log phase and stationary phase *C. thermocellum* DSM 1313 cells were aliquoted anaerobically into 1.5 mL centrifuge tubes. To prepare live cell aliquots without medium, 1 mL of live cells (both log phase and stationary phase) were aliquoted and washed three times with and resuspended in anaerobic distilled water. To prepare spent medium aliquots, 1 mL of log phase cells were aliquoted, centrifuged at 14,000 rpm for 3 min, and filtered with a 0.22-micron syringe filter. All preparations were conducted in duplicate. Methyl viologen was added to all preparations to a final concentration of 150 mg/L, incubated anaerobically at 55 °C for five days and visually inspected for blue coloration as an indication of reduced methyl viologen.

## Additional files

10.1186/s13068-015-0394-9 Batch fermentation performance under methyl viologen and hydrogen peroxide initial loadings.
10.1186/s13068-015-0394-9 Calculated differential expression and adjusted *p* values for genes showing significant (adjusted *p* value < 0.05) differential expression during at least one timepoint of either methyl viologen or hydrogen peroxide exposure.
10.1186/s13068-015-0394-9 (A) Adjusted OD_600_ of batch cultures grown at various initial hydrogen peroxide concentrations. Cultures were grown in MTC media containing 1.1 g/L cellobiose; (B) Chemostat OD_600_ and measured redox potential before, during and after hydrogen peroxide addition; (C) Detailed view of boxed region indicated in panel (B).
10.1186/s13068-015-0394-9 qPCR validation of expression differences of selected genes of interest.

## Abbreviation

CBP: Consolidated BioProcessing
