# Supplementary material for: Clostridium thermocellum DSM 1313 transcriptional responses to redox perturbation
Source: Biotechnol Biofuels. 2015 Dec 12;8:211. doi: 10.1186/s13068-015-0394-9 (PMC4676874; doi:10.1186/s13068-015-0394-9)
Supplement: Supplementary file 1 — 10.1186/s13068-015-0394-9 Batch fermentation performance under methyl viologen and hydrogen peroxide initial loadings. [file 13068_2015_394_MOESM1_ESM.docx]

Additional File 1

Batch fermentation performance under methyl viologen and hydrogen peroxide initial loadings


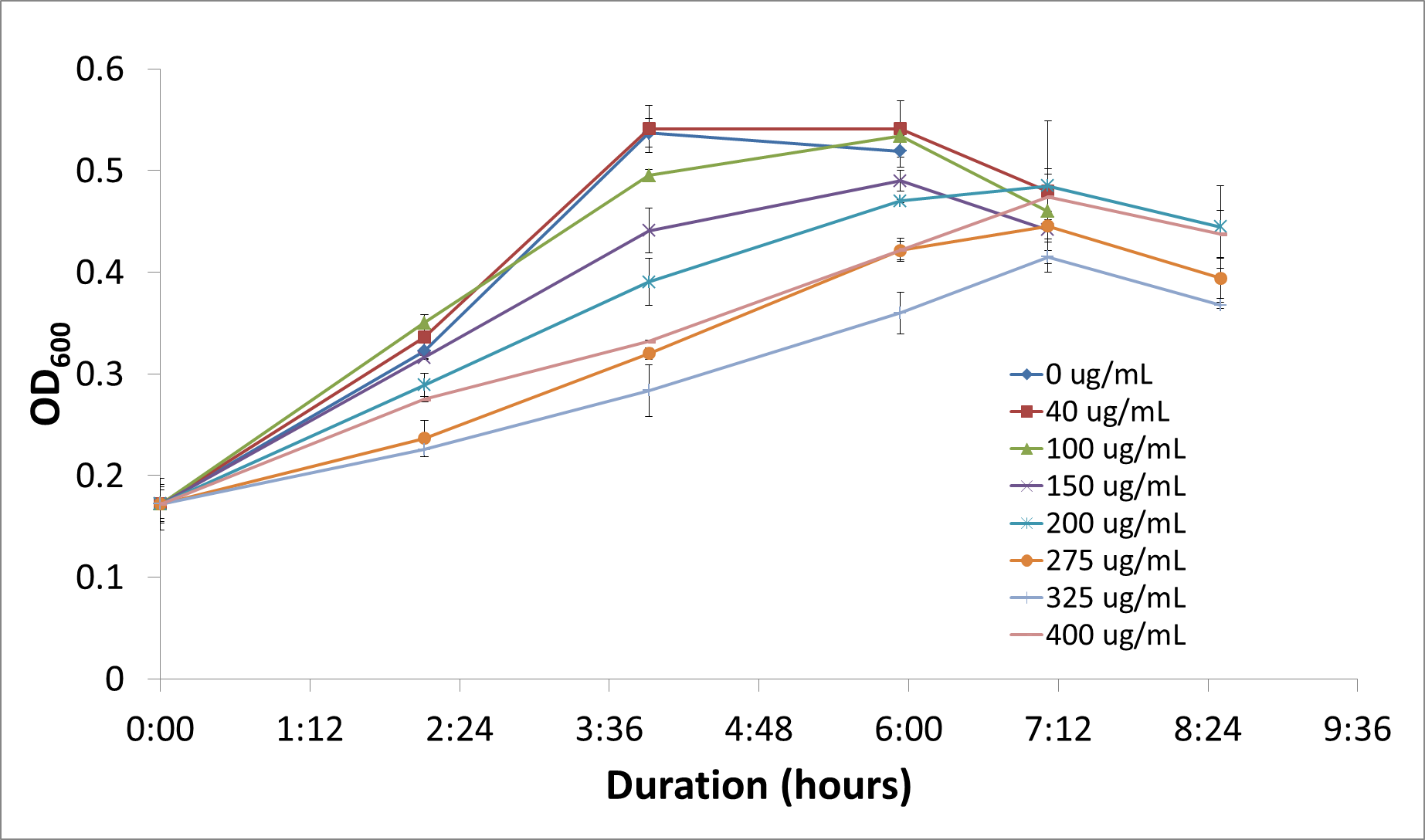


Adjusted OD_600_ of batch cultures grown at various initial methyl viologen concentrtitons. OD_600_ values were adjusted to account for initial blue coloration due to methyl viologen reduction. Cultures were grown in MTC media containing 1.1 g/L cellobiose.


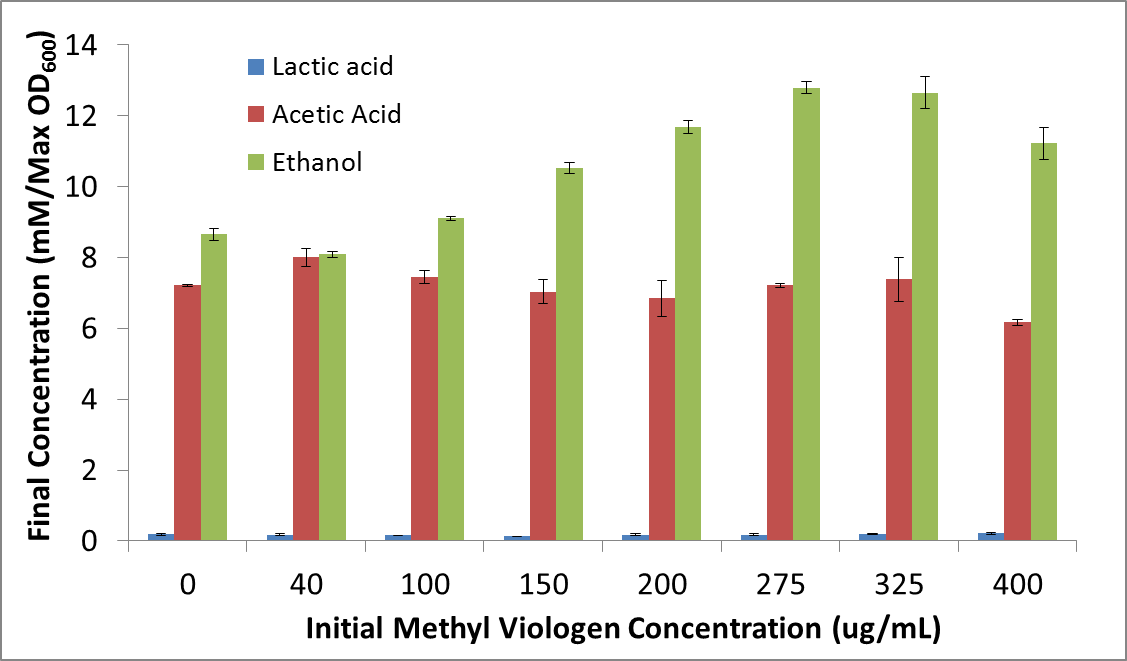


Specific end-point fermentation products at different initial methyl viologen concentrations, normalized to maximum OD_600_ achieved. Cultures were grown in MTC media containing 1.1 g/L cellobiose.


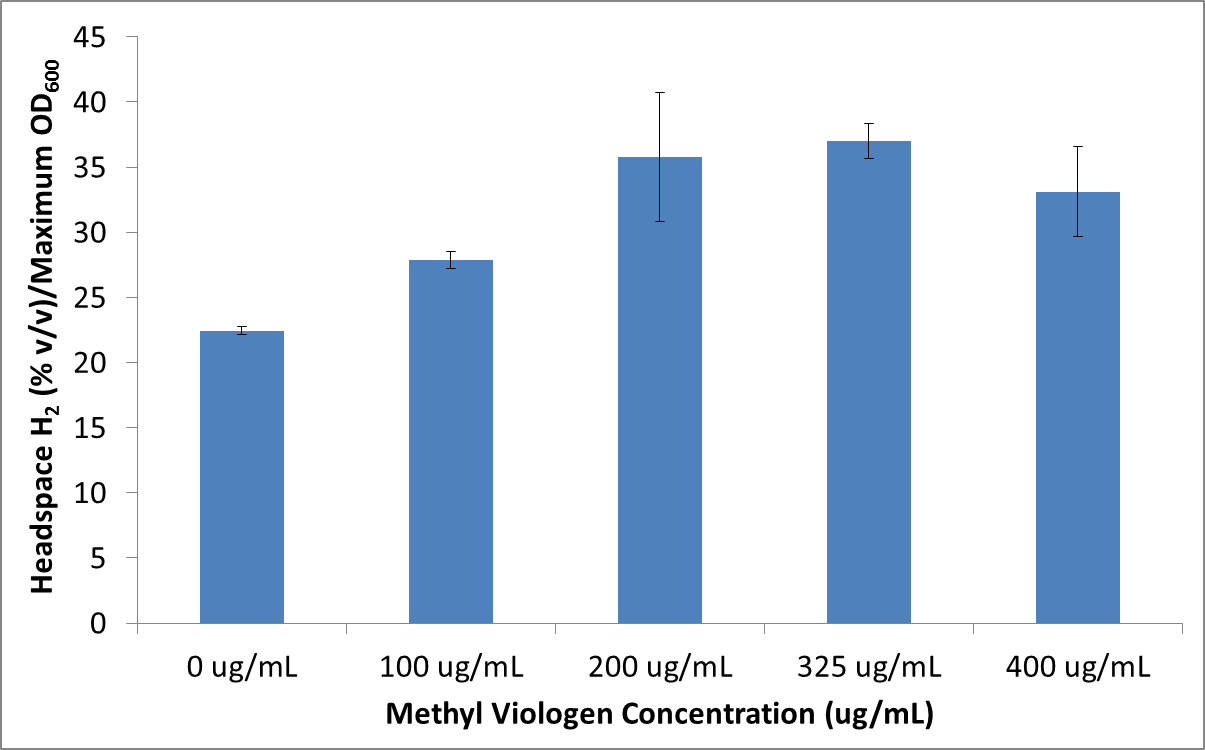


Specific end-point headspace H_2_ at different initial methyl viologen concentrations, normalized to maximum OD_600_ achieved. Cultures were grown in MTC media containing 1.1 g/L cellobiose. Initial headspace % (v/v) H_2_ was 5%.
