# Supplementary material for: Clostridium thermocellum DSM 1313 transcriptional responses to redox perturbation
Source: Biotechnol Biofuels. 2015 Dec 12;8:211. doi: 10.1186/s13068-015-0394-9 (PMC4676874; doi:10.1186/s13068-015-0394-9)
Supplement: Supplementary file 3 — 10.1186/s13068-015-0394-9 (A) Adjusted OD600 of batch cultures grown at various initial hydrogen peroxide concentrations. Cultures were grown in MTC media containing 1.1 g/L cellobiose; (B) Chemostat OD600 and measured redox potential before, during and after hydrogen peroxide addition; (C) Detailed view of boxed region indicated in panel (B). [file 13068_2015_394_MOESM3_ESM.docx]

A.
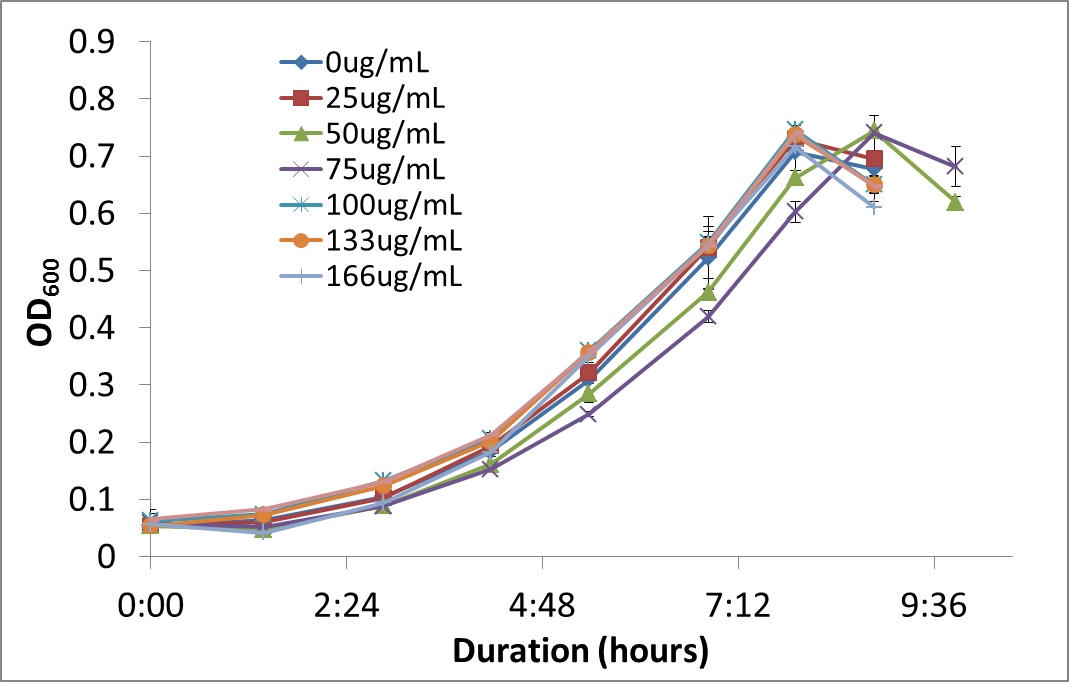


B.
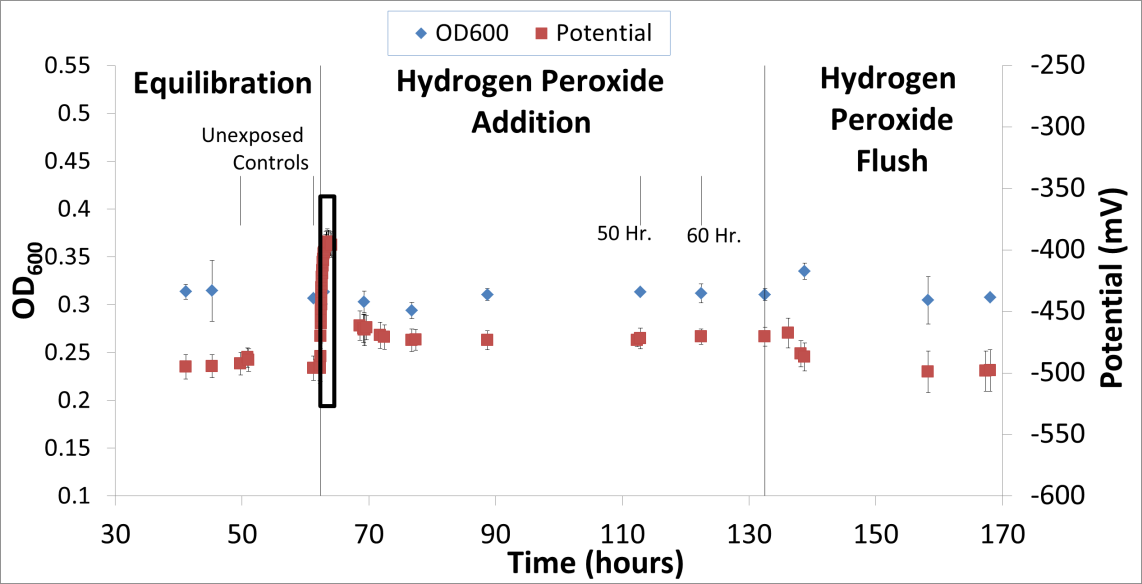


C.
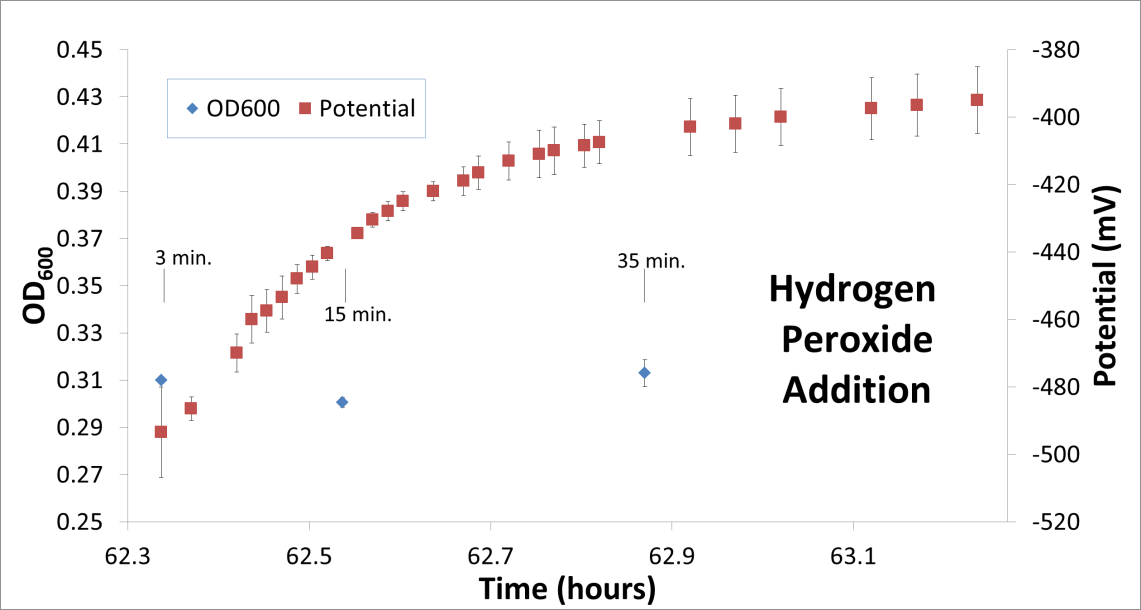


Additional file 3. (A) Adjusted OD_600_ of batch cultures grown at various initial hydrogen peroxide concentrations. Cultures were grown in MTC media containing 1.1 g/L cellobiose; (B) Chemostat OD_600_ and measured redox potential before, during and after hydrogen peroxide addition; (C) Detailed view of boxed region indicated in panel (B).
